# Rate, risk factors and estimations of time to develop severe acute malnutrition after children receiving antiretroviral therapy at selected health facilities in northwest Ethiopia

**DOI:** 10.1017/jns.2023.42

**Published:** 2023-05-22

**Authors:** Dejen Tsegaye, Tsehay Kebede, Fassikaw Kebede

**Affiliations:** 1 Department of Nursing, College of Health Sciences, Debre Markos University, Debre Markos; 2 Department of Geography, College of Social Science, Bahir Dar University, Bahir Dar; 3 Department of Epidemiology, College of Health Sciences, Woldia University, Woldia

**Keywords:** Acute malnutrition, AIDS, Children, Ethiopia, HIV, SAM, AHR, adjusted hazard ratio, CHR, crude hazard ratio, CI, confidence interval, FMOH, Ethiopian Federal Ministry of Health, MUAC, mid-upper arm circumference, IQR, interquartile range, SAM, severe acute malnutrition, NGT, nasogastric intubation for feeding, WFH, weight for height, sd, standard deviation

## Abstract

Severe acute malnutrition (SAM) affects up to 50 % of children with HIV, especially those who reside in resource-constrained healthcare setting like Ethiopia. During subsequent follow-up of children factors related to incidence of SAM after antiretroviral therapy (ART) is set on, however, there is no prior evidence. An institution-based retrospective cohort study was employed among 721 HIV-positive children from 1 January to 30 December 2021. Data were entered using Epi-Data version 3.1 and exported to STATA version 14 for analysis. Bi-variable and multivariable Cox-proportional hazard models were employed at 95 % confidence intervals to identify significant predictors for SAM. According to this result, the overall mean (±sd) age of the participants was found to be 9⋅83 (±3⋅3) years. At the end of the follow-up period, 103 (14⋅29 %) children developed SAM with a median time of 30⋅3 (13⋅4) months after ART initiation. The overall incidence density of SAM was found to be 5⋅64 per 100 child (95 % CI 4⋅68, 6⋅94). Children with CD4 counts below the threshold [AHR 2⋅6 (95 % CI 1⋅2, 2⋅9, *P* = 0⋅01)], disclosed HIV status [AHR 1⋅9 (95 % CI 1⋅4, 3⋅39, *P* = 0⋅03)] and Hgb level ≤10 mg/dl [AHR 1⋅8 (95 % CI 1⋅2, 2⋅9, *P* = 0⋅03)] were significant predictors for SAM. Significant predictors of acute malnutrition were having a CD4 count below the threshold, children who had previously reported their HIV status, and having haemoglobin <10 mg/dl. To ensure better health outcomes, healthcare practitioners should improve earlier nutritional screening and consistent counselling at each session of care.

## Introduction

Human immunodeficiency virus-positive/acquired immunodeficiency syndrome (HIV/AIDS) and malnutrition form a deadly duo with each one fuelling the other. Malnutrition increases susceptibility to infection by causing immune dysfunction in manifold ways. The depressed immune status can amplify HIV replication and accelerate the progression of HIV disease to AIDS stages^(1,2)^. Untreated or advanced HIV/AIDS is again associated with a compromised immune status that makes patients susceptible to lethal opportunistic infections. Rationally, the HIV disintegrates the primary physical defense barriers of collagen levels in skin cells and alters intracellular neovascularisation^(3,4)^. Evidence suggested that inadequate calorie intake due to elevated levels of proinflammatory cytokines like interleukin-1(IL-1), interleukin-6 (IL-6) and tumour necrosis factor-α (TNF-α) can cause anorexia and oxidatively stressed derangements of metabolites^(3,5,6)^.

The clinical presentation of severe acute malnutrition (SAM) includes severe visible wasting (marasmus), nutritional oedema (kwashiorkor) or marasmus–kwashiorkor. However, kwashiorkor and marasmus among HIV-infected children have a higher risk of mortality^(7,8)^. In sub-Saharan Africa, the epidemiology of SAM has increased among children requiring hospitalisation composed of those who are HIV infected, with case fatality rates reaching as high as 20–50 %^(9–11)^. HIV-infected children with SAM are more likely to present with other comorbidities and complications (e.g. TB, severe anaemia, persistent diarrhoea or villous atrophy and disaccharide intolerance), which contribute to loss of appetite, poor oral intake and continual lean body mass^(7,12)^.

Globally, in 2019, 1⋅7 million children are infected with the HIV; in the same year, 44 229 children in Ethiopia were infected with HIV, and 2055 died because of AIDS^(5,7)^ and case fatality rates among children with SAM are often as high as 20–30 %^(13)^. The greatest burden of malnutrition is seen in the peak age group of 2–5 years^(1,14)^. HIV increased poor nutrition as a result of poor food intake and fuelled nutrient usage from the body increases the incidence of lethal opportunistic infection for further hospitalisation^(15)^. A systematic review and meta-analysis on children with SAM in sub-Saharan Africa revealed that children with HIV infection were more likely to die than those not infected with HIV (30⋅4 *v.* 8⋅4 %)^(6,10,11)^. When weight loss was >10 % below the baseline weight, the relative risk of death increased nearly six-fold^(1,5,16)^. In addition, even relatively small losses in weight (5 %) are associated with a decrease in the survival rate of HIV-infected children.

According to the Health and Health-Related Indicators (HHRIs) of 2014, SAM was the third leading cause of mortality (accounting for 8⋅1 % of deaths) and 20 % of hospital admissions for children in Ethiopia^(17–19)^ and mortality rate in HIV children undergoing SAM treatment is found to be 5⋅4 per 100 child-years in northwest Ethiopia^(20)^. A major contribution to the excess mortality due to malnutrition in HIV-positive children is the inaccessibility of timely and appropriate medical services, which are plentiful and vary at regional and district levels^(21)^. Financial fees for accessory transport and treatment cost, which are frequent barriers to paediatric healthcare services in many developing countries including Ethiopia^(16,21)^. Even though SAM is a leading cause of hospitalisation and mortality for HIV-infected children, there is no prior evidence in Ethiopia on when to develop SAM and associated risk factors for seropositive children after antiretroviral therapy (ART) is initiated. Hence, the present study aimed to estimate the half-life time to develop SAM in HIV-infected children treated with ART care in selected health facilities of the Metekel Zone including Assosa Hospital, Northwest Ethiopia.

## Methods

### Study setting and time

The present study was conducted in the Metekel Zone at a selected health facility from 1 January 2011 to 30 December 2021. The Metekel Zone is one of the three administrative zones, located in the Benishangul Gumuz region. The region has two referral hospitals, three primary hospitals and more than thirty-two health centres. The present study was conducted in two health centres and two referral hospitals. The main reason including only four health institutions is earlier endorsement and initiation of ART care for more than 2968 catchment adult and children population received ART care^(22)^.

### Study design

A facility-based retrospective cohort study was conducted.

### Source population

The source population was the entire HIV-positive children who had enrolled in HIV/AIDS chronic care in selected health facility in northwest Ethiopia.

### Study population

All HIV-positive children enrolled and started ART in selected health facility from 1 January 2011 to 30 December 2021.

### Inclusion criteria

All HIV-positive children (≤15 years of age) were enrolled for HIV/AIDS chronic care without SAM at baseline from 1 January 2011 to 30 December 2021 in selected health facility.

### Exclusion criteria

Children at baseline registered for ART and whose ART follow-up care did not contain variables including age of children and date of ART initiation were excluded.

### Study population

All HIV-positive children who started taking ART between 1 January 2011 to 30 December 2021, at two health centres and two referral hospitals qualified as study participants and were eligible for the final analysis.

### Sample size determination

The sample size was calculated by using log-rank survival data analysis of the double population proportions formula by using the following assumption, 95 % confidence level, 80 % optimum statistical power and taking one error of 5 %. Considering a study that was conducted in the same place in northwest Ethiopia and taking rural residents as a predictor variable for the exposed group of seropositive children denoted by q1 (0⋅52) and urban residents not exposed group denoted by q0 (0⋅48)^(5)^. After adding 10 % incomplete medical records, the final sample size was found to be 630. Nevertheless, since children ≤15 years enrolled for ART care in two hospitals and health centres from 1 January 2011 to 30 December 2021 were found to be 732. Hence, all study participants were included, and all study participants without a sampling procedure due to increase statistical power.

Finally, since children under the age of 15 were enrolled for ART care in two hospitals and health centres from 1 January 2011 to 30 December 2021, and after we had retrieved seropositive children's ART follow-up cards and screened for eligibility requirements (11 files are removed), we included 721 individual files without any sampling procedure to increase statistical power.

### Dependent variable

Incidence of severe acute malnutrition.

### Independent variables

Socio-demographic characteristics of the children (age, sex, residence, family size, caregiver, parental status), baseline clinical and laboratory factors, characteristics like ART regimen, functional status, developmental status, nutritional status, and opportunistic infections, and follow-up factors (level of ART adherence, CPT, IPT, TB contact history and vaccination status).

### Operational definitions

*Events:* Children with under-fifteen HIV-positive children who had an admission history of SAM (MUAC <11⋅5 cm, or weight-for-height/length *Z*-score <−3, or bilateral pitting oedema of other causes excluded with or without severe wasting *Z*-score <−3) were an event for this study. *Censored:* If the child had lost follow-up or transferred out to another service before developing SAM, or if the child was free from SAM until the end of our data collection day.

*Adherence to ART:* ART was classified based on the percentage of drug dosage calculated from the total monthly doses of ART drugs (Good > 95 %, fair 85–94 % and poor < 85 %)^(18)^.

*Anaemia:* It was defined as having a haemoglobin level ≤10 mg/dl^(23)^.

*CD4 count:* CD4 levels below the threshold level were classified based on the child's age (i.e. infants CD4 1500/mm^3^, 12–35 months 750/mm^3^, 36–59 months 350/mm^3^ and 5 years 200/mm^3^)^(24)^.

*Time to SAM:* Newly diagnosed of SAM in HIV-positive children after ART initiated; *Severe acute malnutrition:* which is defined by the WHO as a weight-for-height *Z*-score of less than −3 *Z*-curve, or a mid-upper arm circumference of less than (MUAC) < 11⋅5 cm in a child aged 6 months to 5 years, or the presence of bilateral oedema with failed appetite test^(5)^.

*CD4 count:* CD4 levels below the threshold level were classified based on the child's age (i.e. infant CD4 1500/mm^3^, 12–35 months 750/mm^3^, 36–59 months 350/mm^3^ and 5 years 200/mm^3^)^(25)^.

*Incomplete data:* Children at baseline registration for ART, whose ART follow-up care did not contain variables including age of children, and ART initiation date.

### Data collection instruments and quality control

Standard and pretested data extraction tools were used to extract the required information from the case notes both for new and readmitted cases. Before the actual data collection, the prepared checklist of variables was pretested in thirty-seven case notes of HIV-infected children at Jawi Primary Hospital. Two-day training was given for two diploma nurse data collectors and for a degree public health officer about the objective of study outcome and importance of maintaining data confidentiality.

### Data processing and analysis

After coding, data were entered into Epi-Data version 4.2, and then exported to STATA (se)/14 for further analysis. The WHO Anthro-Plus-Version 1.04 and ENA for Nutrition Smart Software were used to generate the *Z*-score (WAZ, HAZ and WHZ/BAZ) to define the nutritional status of HIV-positive children. The incidence rate of SAM is calculated using the total number of people per year (PPY) individual contribution to follow-up as a denominator. Descriptive nonparametric statistical tests such as the Kaplan–Meier plot were used to estimate the median SAM-free survival time. Assessing whether a real statistically significant survival difference between the two groups was tested by using the log-rank test.

The necessary Cox-proportional hazard model assumption was checked using a graphical diagnostic based on the scaled Schoenfeld residuals (log–log survival plot) and statistical tests (using the global test estimations). The multivariable Cox-proportional hazard regression for this study was written during consecutive follow upon HIV-infected children individuals under observation experiencing the event (SAM) in a period centred on that point in time. The covariates of the multivariate analysis were selected using the enter method. Variables with *P*-value <0⋅2 in the bi-variable Cox regression analysis were included in the multivariable Cox regression model to determine the factors associated with SAM incidence. Deviance Information Criteria (DIC), Akaike's Information Criterion (AIC) and Bayesian Information Criterion (BIC) are used to compare the candidate Cox-proportional hazard to the checked final model fitted. The model with *P*-value less than 0⋅05 will be selected as the final model of the analysis and checked using Nelson-Aalen and Cox-Snell residual tests (Fig. 6).

## Results

### Socio-demographic characteristics of HIV-infected children

Overall, 732 children living with HIV files were reviewed for this study. However, eleven individual charts (1⋅6 %) were excluded due to incompleteness. The majority 384 (53⋅26 %) of the respondents were female in gender, and 510 (70⋅74 %) of those were urban inhabitants. The overall mean (±sd) age of participant children was found to be 9⋅83 (±3⋅3) years. A large proportion of 381 (52⋅84 %) children had lived with parents (Table 1).

**Table 1.** Socio-demographic characteristics of children living with HIV in the Metekel Zone at selected health facilities in northwest Ethiopia from 1 January to 30 December 2021 (*n* 721)

Variables	Categories	Frequency	Percent (%)
Age of children	≥5 years	78	10⋅8
	6–10 year	254	35⋅2
	11–15 years	389	53⋅9
HIV disclosure status of children	Disclosed	158	21⋅9
	Not disclosed	563	78⋅1
Age of primary caregiver	≤45 years	244	38⋅8
	<45 years	477	66⋅1
Marital status of the primary caregiver	Single	115	15⋅9
	Married	498	69⋅1
	Divorced	82	11⋅4
	Widowed	26	3⋅6
Family size of primary caregivers	≤2	227	31⋅4
	3–5	462	64⋅1
	≥6	32	4⋅4
HIV status of primary caregiver	Positive	550	76⋅3
	Negative	91	12⋅6
	Unknown	80	11⋅1
Religions of primary caregiver	Orthodox	381	52⋅8
	Muslim	152	21⋅2
	Protestant	139	19⋅3
	Catholics	49	6⋅8
Occupational status of primary caregiver	Farmer	99	13⋅8
	Merchant	337	46⋅7
	Employer	124	17⋅2
	Labourer worker	161	22⋅3
Parental status of primary caregiver	Both alive	381	52⋅8
	Paternal orphan	135	18⋅2
	Maternal orphan	108	14⋅9
	Both orphaned	97	13⋅5

### Baseline clinical and comorbidity characteristics

More than half 487 (67⋅47 %) of the participant children had PMTC follow-up during pregnancy and almost all 467 (94⋅47 %) of the caregivers in their dyads had nutritional counselling at the baseline. Nevertheless, 86 (11⋅93 %), 139 (19⋅2 %) and 70 (9⋅9 %) of those children developed severe wasting, stunting and being underweight, respectively. The majority (57⋅3 %) of the participant children had a CD4 count above the threshold, but nearly two in five 287 (39⋅8 %) of those were in CLINICAL stages III&IV. Nearly half 302 (41⋅6 %) of the participant children did not receive CPT, 270 (37⋅4 %) did not receive IPT, 510 (70⋅7) had no ART regimen change and 463 (64⋅2 %) had no opportunistic infection. At the baseline, the mean (±sd) weight and MUAC of participant children were found to be 11⋅3 (±23⋅6) kg and 10⋅8 (±1⋅4 cm), respectively (Table 2).

**Table 2.** Baseline clinical and immunological characteristics of seropositive children in selected health facilities in northwest Ethiopia from 1 January to 30 December 2021 (*n* 721)

Variables	Categories	Frequency	Percent (%)
Functional status (age ≤ 5 years)	Appropriate	69	71⋅9
	Delay	15	15⋅6
	Regression	12	12⋅5
Developmental history (age >5 years)	Working	488	77⋅9
	Ambulatory	87	13⋅9
	Bedridden	51	8⋅15
Adherence	Good	356	49⋅4
	Fair	177	24⋅5
	Poor	188	26⋅1
WHO clinical stage	I	237	32⋅8
	II	202	28⋅1
	III	170	23⋅6
	IV	112	15⋅5
CD4 count or percent	Below threshold	308	42⋅7
	Above threshold	413	57⋅3
Haemoglobin level	≤10 g/dl	229	31⋅7
	>10 g/dl	492	68⋅2
Duration on ART	≤36 months	223	30⋅9
	36–72 months	295	40⋅9
	≥72 months	203	28⋅1
Current status of children	On follow-up	539	74⋅7
	Transferred out	68	9⋅4
	Lost from follow-up	13	1,8
	Drop	11	1⋅5
	Died	90	12⋅4
MUAC	≤11⋅5 cm	270	37⋅4
	>11⋅5 cm	451	62⋅5
Underweight (WFA)	Normal	491	68⋅1
	WAZ < −2 *Z*-score	144	19⋅9
	WAZ < −3 *Z*-score	86	11⋅9
Stunting (HFA)	Normal	428	59⋅4
	HAZ < −2 *Z*-score	154	21⋅4
	HAZ < −3 *Z*-score	139	19⋅2
WFH (Wasting)	Normal	531	73⋅5
	WHZ < BAZ−2 *Z*-score	119	16⋅6
	WHZ or BAZ < −3 *Z*-score	71	9⋅9

### Time to develop SAM in HIV-infected children

A nine-year retrospective cohort study of 721 participant children yielded 21 685 months. The median time to develop SAM was found to be 30⋅3 (IQR ± 13⋅4) months with the crude incidence of SAM at 14⋅2 %. During the follow-up period, about 612 (84⋅88 %) children were on follow-up, while 39 (5⋅4 %) died, 58 (8⋅04 %) lost from follow-up and 12 (1⋅67 %) were transferred out. The median survival probability for participant children was found to be 89⋅76 % (86⋅74; 92⋅12). The overall incidence density rate of SAM was 5⋅64 per 100 child-year observations (95 % CI 4⋅68, 6⋅94).

**Fig. 1.**
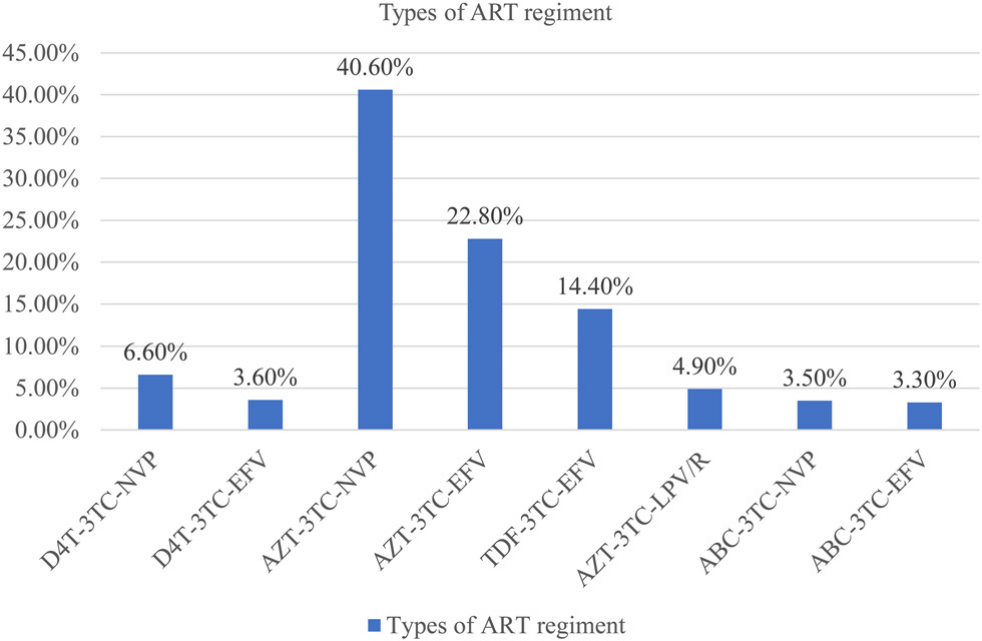
Types of ART regimen for children receiving ART at selected health facilities in northwest Ethiopia from 1 January to 30 December 2021 (*n* 721).

**Fig. 2.**
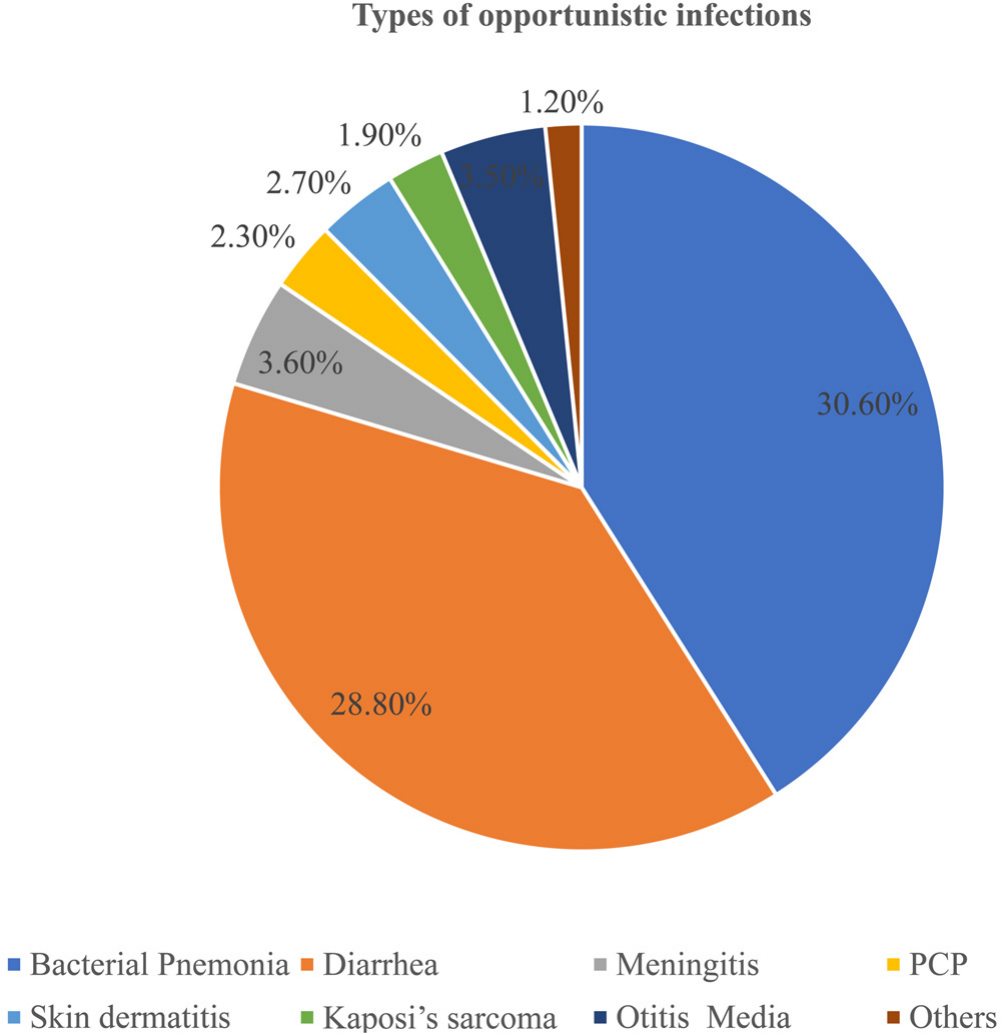
Types of opportunistic infections for children receiving antiretroviral therapy at selected health facilities in northwest Ethiopia from 1 January to 30 December 2021 (*n* 721).

### Kaplan–Meier survival of SAM

**Fig. 3.**
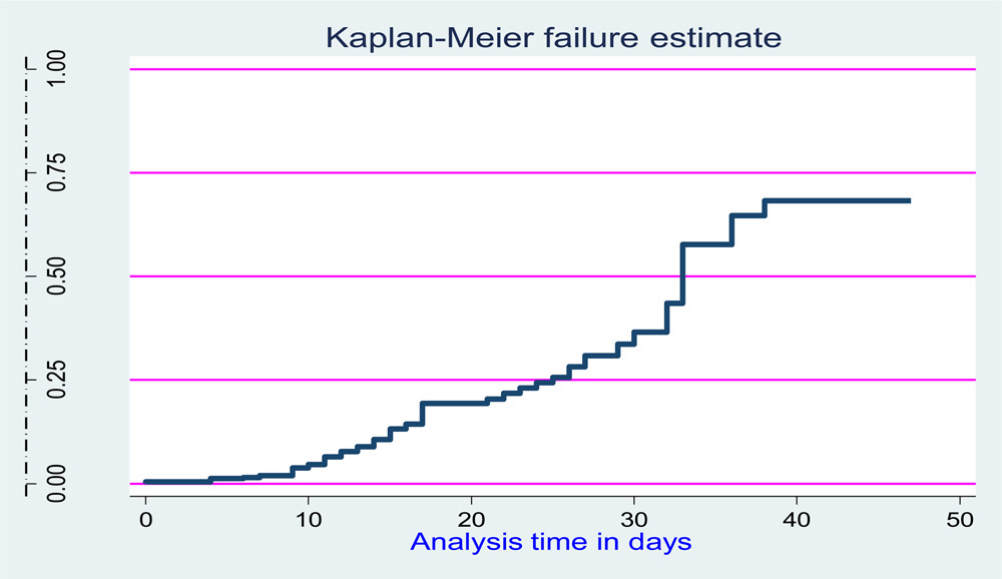
Overall hazard-estimate of attrition for under-five children after started inpatient treatment for complicated SAM at Pawe general hospital in northwest Ethiopia from 1 January to 30 December 2021 (*n* 721).

There was a significant survival difference in the time of developing SAM among the categorical variables like below threshold CD4 count disclosed HIV status, and levels of haemoglobin at the baseline had a significant survival difference (Fig. 3).

**Fig. 4.**
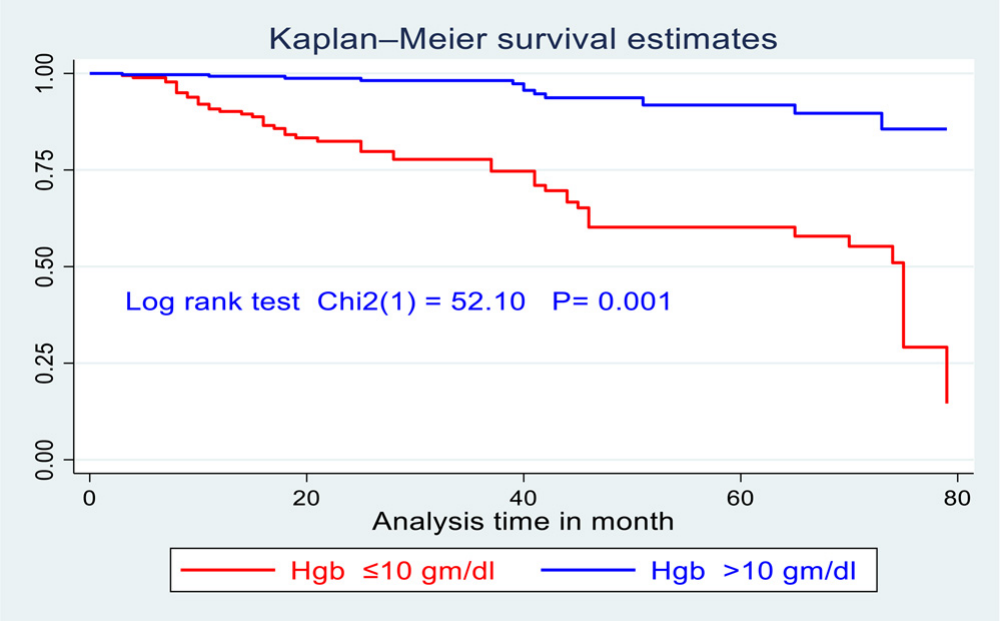
Kaplan–Meier survival curve of children living with HIV stratified by levels of haemoglobin who enrolled for ART in northwest Ethiopia from 1 January to 30 December 2021 (*n* 721).

Accordingly, there was a significant survival difference in the incidence of SAM between children who had haemoglobin ≤10 gm/dl as compared with those who had haemoglobin levels > 10 gm/dl and evidence from the log-rank test (Chi2(1) = 52⋅1, *P* = 0⋅001) (Fig. 4).

**Fig. 5.**
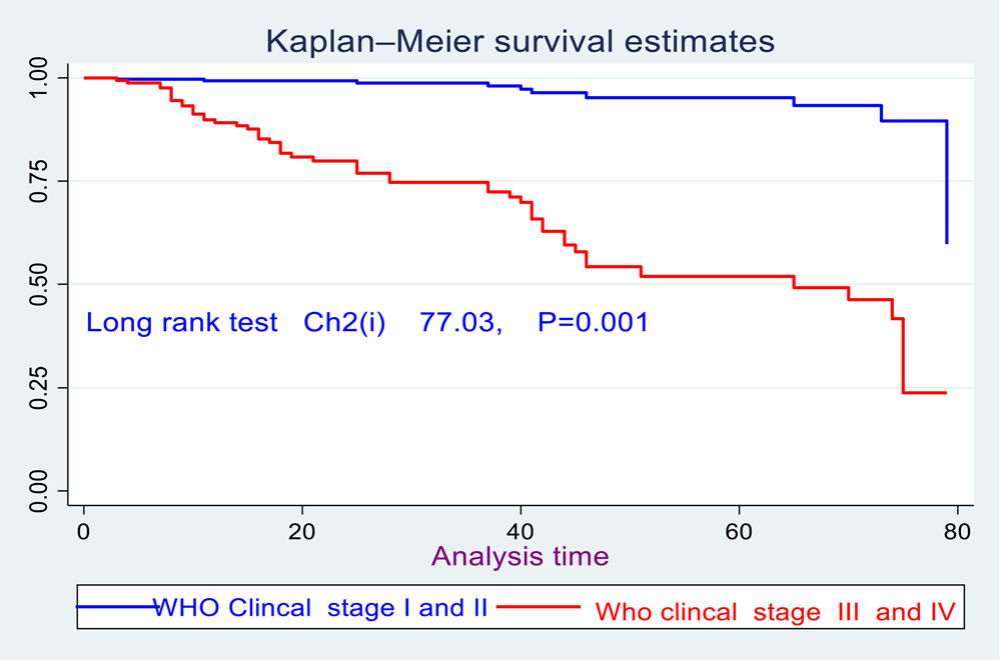
Kaplan–Meier survival curve of children living with HIV stratified by WHO clinical stages in enrolled for ART care in northwest Ethiopia from 1 January to 30 December 2021 (*n* 721).

Furthermore, there was also a significant survival difference for children who had WHO clinical stages III&IV at the baseline as compared with those who had children WHO clinical stages I&II; which is evidenced by the log-rank test (Chi2(1) = 77⋅1, *P* = 0⋅001) (Fig. 5).

### Predictors for time to develop SAM in HIV-infected children

During bi-variable *Cox regression* analysis, variables were checked whether they were factors associated with time to develop SAM at *P*-value <0⋅2 for a candidate transferee of multivariable *Cox regression*. After adjusting certain confounding, eleven variables were fitted to build the final model with three independent factors associated with the incidence of SAM. The risk of SAM among children with CD4 counts below the threshold level was 2⋅6 increased as compared with those child having CD4 counts above the threshold [AHR 2⋅6 (95 % CI 1⋅2, 2⋅9, *P* = 0⋅01)]. Likewise, the risks of SAM for HIV-positive children with disclosed their HIV status were 1⋅9 times the increased risk of developing SAM as compared with those children not disclosed HIV status [AHR 1⋅9 (95 % CI 1⋅4, 3⋅39, *P* = 0⋅03)]. Moreover, the risk of developing SAM for baseline levels of haemoglobin ≤10 mg/dl is nearly two [AHR 1⋅8 (95 % CI 1⋅2, 2⋅9, *P* = 0⋅03)] times increased as compared with the counter group having >10 mg/dl at the baseline (Table 3).

**Table 3.** Bi-variable and multivariate Cox regression analysis for time to develop SAM among HIV-infected children in northwest Ethiopia from 1 January to 30 December 2021 (*n* 721)

Variables	Categories	Survival status		AHR	*P*-value
		Event	Censored		
Age of children	≤5 years	57	83	1⋅4(0⋅75–2⋅4)	0⋅24
	6–10	25	229	1⋅2(0⋅33–2⋅1)	0⋅32
	11–15	21	306	Ref	
Sex	Male	35	302	Ref	
	Female	56	328	1⋅7(0⋅7–2⋅6)	0⋅28
Residence	Rural	61	449	1⋅2(0⋅6–1⋅85)	0⋅63
	Urban	29	182	Ref	
CD4 count	Below threshold	70	238	2⋅6(1⋅2–2⋅85)	0⋅01
	Above threshold	20	393	Ref	
WHO stage	Stages I&II	33	477	Ref	
	Stages III&IV	70	141	1⋅8(0⋅94–3⋅39)	0⋅12
Adherence	Good	35	463	Ref	
	Fair/poor	68	155	1⋅7(0⋅6–1⋅9)	0⋅35
Dietary counselling	Yes	62	403	Ref	
	No	41	215	1⋅2(0⋅82–1⋅9)	0⋅29
Disclosure status	Yes	50	108	Ref	
	No	53	510	1⋅6(1⋅4– 3⋅4))	0⋅03
Treatment failure	Yes	24	79	1⋅26(0⋅71–2⋅1)	0⋅22
	No	20	598	Ref	
Levels of haemoglobin	≤10 mg/dl	76	154	1⋅8(1⋅2–2⋅9)	0⋅03
	>10 mg/dl	27	464	Ref	

### Overall, the model adequacy test

**Fig. 6.**
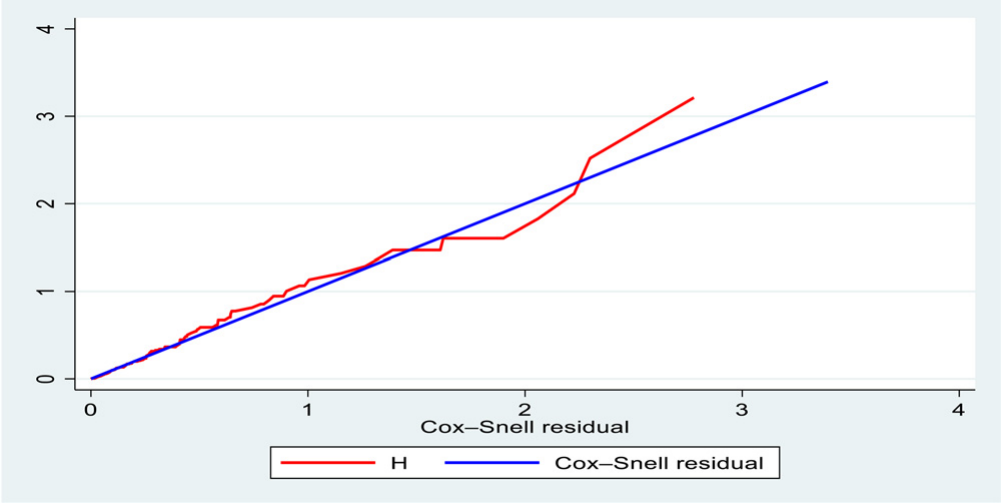
Nelson-Aalen and Cox-Snell residual tests for the final model adequacy test.

The overall multivariable Cox regression test of model adequacy for SAM incidence in HIV-positive children after ART started and it indicates that the line is on the straight origin (Fig. 6).

## Discussion

At the end of the study period, 103 (14⋅2 %) participants’ children developed SAM with maximum and minimum time to develop between 3 and 98 months. This finding is lower than previously reported studies done in south Gondar hospital (26⋅72 %)^(7)^, East Africa (24⋅65 %)^(10)^, sub-Saharan Africa (24⋅5 %)^(26)^, West Africa (26 %)^(27)^, Malawi (17⋅5 %)^(4)^ and Burkina Faso (63 %)^(2)^. This might be due to the difference in quality care, cut-off point for SAM diagnosis and ability of healthcare providers to screen in different health institutions^(24)^. Likewise, the overall incidence density rate in our study was found to be 5⋅64 per 100 PPY (95 % CI 4⋅68, 6⋅94) and is comparable with previously reported studies in 5⋅4 per 100 PPY at Debre Tabor^(7)^ and 5⋅42 per 100 PPY in Pawe hospitals^(5)^. This might be due to the difference in socio-demographic characteristics, study population and study design^(7)^. In contrast to the 126-month finding in the Debre Tabor Hospital, the median time to develop SAM in our report was found to be 30⋅3 months. This could be explained by the quality of treatment and screening capabilities of the healthcare workers in the two institutions. Given that Debre Tabor Hospital is a specialised university hospital, our study setting consists of health centres and general hospitals^(7,25)^.

Regarding predictors of SAM, the risk of SAM among children with a baseline CD4 count below the threshold level was three times increased than that of children with a CD4 count above the threshold levels [AHR 2⋅6 (95 % CI 1⋅2, 2⋅9)]. This is comparable with findings reported in Pawe Hospital^(5)^, Debre Tabor Hospital^(7)^, Burkina Faso^(28)^, Nigeria^(29)^, Malawi^(4)^ and Asia Pediatric HIV Observational Database^(26)^. This might be because children who have low CD4 count can be exposed to chronic diarrhoea, tuberculosis and lethal opportunistic infections, which ought to cause a significant imbalance in nutritional demand and individual intake both in quantitative (number of kilocalories/day) and qualitative (vitamin and minerals, etc.) deficiencies. What is more, the proinflammatory cytokines like interleukin-1 (IL-1), interleukin-6 (IL-6) and tumour necrosis factor-α (TNF-α) for a long time cause anoxia and lack of interest in feeding^(3,5,6)^, this might expose for metabolic derangement due to oxidative stress and hasten viral replication in progressions of AIDS disease^(16,30)^.

Furthermore, compared to their not disclosed HIV status, children that disclosed they were HIV positive had a nearly two-fold higher risk of developing SAM [AHR 1⋅9 (95 % CI 1⋅4, 3⋅39)]. This might be due to the effect of disclosing HIV status for children ≤15 years. Disclosure of a child's HIV status to the child has value in terms of positive health outcomes such as better adherence and slower disease progression. However, it has negative health consequences on increased psychiatric hospitalisation^(31)^, and prolonged loss of appetites with a marked weight reduction loss^(24)^, all of these further push children towards nutritional impoverishment.

Consistent with previous findings at worksite hospital^(1)^, south wall Hospital^(32)^, Pawe Hospital^(25)^, Deber Tabor Hospital^(7)^, Western Kenya^(6)^ and Burkina Faso^(2)^; the risk of developing SAM after ART for seropositive children with Hgb ≤10 mg/dl were nearly two times increased as compared with counter group [AHR 1⋅8 (95 % CI 1⋅16, 2⋅9)]. A consistent report on earlier research suggested that HAART regimens containing zidovudine (ZDV) are a substantial contributor to severe anaemia, particularly in children with HIV^(33)^. Reduced synthesis of red blood cells (RBCs) and increased RBC oxidation are two ways this is shown with this HAART regimen and malnutrition will therefore inevitably increase along with the phase of HIV disease.

### Limitations

The present study had inherent limitations resulting from its retrospective study design; however, missing significant variables like caregivers’ household economic assets and lack the educational status in the recoded files might bias final interpretations.

## Conclusion

Significant predictors of acute malnutrition were having a CD4 count below the threshold, children who had previously reported their HIV status, and having haemoglobin <10 mg/dl. To ensure better health outcomes, healthcare practitioners should improve earlier nutritional screening and consistent counselling at each session of care.
